# Adult Childrens’ College Completion Timing and Their Aging Mothers’ Self-Rated Health: The Role of Mothers’ Educational Attainment

**DOI:** 10.1007/s10804-024-09484-9

**Published:** 2024-06-01

**Authors:** Xing Zhang, Anna M. Hammersmith, Masumi Iida, Frank Infurna

**Affiliations:** 1 College of Health Solutions, Arizona State University, 425 N. 5th Street, Phoenix, AZ 85004 USA; 2 Department of Sociology, Grand Valley State University, Allendale, MI USA; 3 T. Denny Sanford School of Social and Family Dynamics, Arizona State University, Tempe, AZ USA; 4 Department of Psychology, Arizona State University, Tempe, AZ USA

**Keywords:** Intergenerational relationships, Educational attainment, Aging and the life course

## Abstract

The transition to adulthood has become delayed, with college completion often diverging by socioeconomic background, specifically maternal education. On time, late, or never completing college can have health ramifications that reverberate into the lives of aging mothers. Using dyadic data from Waves I, III, IV, and V of Add Health and Waves I and II of Add Health Parent Study, we used logistic regression to assess whether the adult childrens’ college timing completion was associated with their aging mothers’ self-rated health. We also considered variation by mothers’ educational attainment. Results showed adult childrens’ college completion, whether on time or late, was associated with better maternal self-rated health relative to having adult children who did not complete college. We found no evidence that college completion timing and mothers’ self-rated health varied by mothers’ educational attainment.

## Introduction

Adulthood is a “demographically dense” period of life in which adult children complete key milestones such as finishing college, finding employment, marrying, and entering parenthood (Rindfuss, 1991). Adult milestones are often accompanied by societal timing expectations, or anticipated ages when markers should be achieved (Cepa & Furstenberg, 2021). If adult milestones are not achieved “on time,” there may be health consequences. In fact, a growing body of work underscores how an adult child’s achievements, like college completion, can reverberate into the lives of others, including their midlife or aging mother (hereafter referred to as ‘aging mother’), shaping mothers’ health and well-being (Friedman & Mare, 2014; Lee et al., 2017; Oreopoulos & Petronijevic, 2013; Peng et al., 2019; Yahirun et al., 2017, 2020).

Although completing college is considered one important marker of adulthood in the U.S., earning a college degree has shifted later into adulthood (Cepa & Furstenberg, 2021). Today, adult children devote more time to higher education, with about 48% of students completing college in four years, and fewer than two-thirds finishing a degree in six years. Such delays push an adult child’s college completion into later adulthood (2022b; NCES, 2022a). Given the importance of mother–child bonds, shifts in adult children’s college completion timing may shape health for their aging mothers. College delayed or forgone may increase adult childrens’ support needs from their aging mother. Mothers may also worry about adult children who do not complete college on time, creating stress, thereby harming health. In this paper, we explore differences in self-rated health (hereafter health) of mothers who have adult children complete college on time relative to late or never. We also consider variation by mothers’ educational attainment (specifically if they completed college), as expectations surrounding college completion and the link to health likely vary by socioeconomic background (Cepa & Furstenberg, 2021; Cooper & Pugh, 2020).

Using Waves I, III, IV, and V of the National Longitudinal Study of Adolescent to Adult Health and Waves I and II of Add Health Parent Study (hereafter Add Health and AHPS, respectively), we employ logistic regression to assess whether college completion (e.g., on time, late, or never) by adult children is associated with their mothers’ health. This study offers several major contributions to research on adult childrens’ educational attainment and health of aging mothers. Although several studies have revealed a link between an adult child’s college completion and mothers’ health, none to date have used data on both members of the mother–child dyad (Friedman & Mare, 2014; Lee et al., 2017; Oreopoulos & Petronijevic, 2013; Peng et al., 2019; Yahirun et al., 2017, 2020). Pairing Add Health and AHPS data allows us to track adult childrens’ college completion reports with mothers’ reports of their own health, reducing reporting bias (Comola & Fafchamps, 2017). Moreover, Add Health permits an examination of adult child’s college completion timing (e.g., on time, late, or never). Few studies consider timing implications when investigating the link between adult childrens’ college completion and their mothers’ health. Finally, we advance prior work by considering how socioeconomic background (operationalized by mothers’ educational attainment) may yield divergent pathways in college completion timing, playing a role in mothers’ own health assessment.

### Life Course Perspective: Linked Lives and Timing

This study draws on life course perspective, focusing on linked lives and timing to better understand how an adult child’s college completion timing may shape their mothers’ health. Life course perspective emphasizes how the lives of mothers and adult children are interdependent, defined by *linked lives* (Elder, 1998). Life course perspective also underscores lifelong development of close ties, highlighting how relationships—like that between a mother and child—change over time (Elder et al., 2004). To illustrate, mothers provide instrumental and emotional support to children from infancy into their young adult years (Rossi & Rossi, 1990). As children enter adulthood, they often acquire independent roles, thereby requiring less support from their aging mothers. Thus, assistance from mothers often tapers off as children age and achieve adult markers, such as finishing college (Rossi & Rossi, 1990).

Life course perspective also highlights the importance of *timing* or expectations of when children should acquire adult roles such as completing college (Cepa & Furstenberg, 2021). Although considered pivotal to adulthood, college completion timing has shifted later into one’s adult years. Educational achievements in the United States are often symbolic of adulthood with over half of Americans citing college as “very important” to success (Marken, 2019). Young adults today are also increasingly college-educated; about 40% of 25–37-year-olds reported a college degree relative to 25% of Boomers and 29% of Generation X when compared at the same age (Bialik & Fry, 2019). Yet, young adults today devote more years to complete higher education either because they enter college later or spend more years enrolled. To illustrate, 48% of students today finish a degree within four years and fewer than two-thirds earn a bachelor’s degree within six years (2022b; NCES, 2022a). Even as timing related to college completion shifts, if adult markers are not achieved “on time,” there may be health consequences not only for the adult child, but also their aging mother.

### Adult Childrens’ College Completion and Their Mothers’ Health

College completion is one of many key adult achievements in the U.S. (Cepa & Furstenberg, 2021). Completing college is a positive development in the life of an adult child, and likely has ramifications for the mother’s health. Further, completing college may yield better health (Walsemann et al., 2012), which could spill over to the aging mother. In this study, we focus on mothers’ self-rated health, as research indicates it is linked to mortality and captures subjective and objective aspects of health (Jylhä, 2009). Several mechanisms explain how childrens’ college completion may be tied to their mothers’ health (Frase et al., 2022), specifically through the transmission of material and non-material resources.

Education can be a source of material resource transmission between adult children and mothers. Adult children who complete college not only become less reliant on their mothers, but they likely have more tangible, material resources to share that could protect their mothers’ health (Oreopoulos & Petronijevic, 2013). However, adult children who do not complete college may challenge norms surrounding the flow of intergenerational support. Mothers often provide financial, emotional, and instrumental support to children into adolescence, with support waning in young adulthood (Rossi & Rossi, 1990). Adult children who do not complete college may need more support from mothers longer into their adult years (Oreopoulos & Petronijevic, 2013).

Education is also a source of non-material resource transmission between adult children and mothers. For instance, adult children who complete college are more likely to encourage mothers to stop smoking, exercise more frequently, eat healthier, and visit the doctor regularly (Friedman & Mare, 2014; Lee et al., 2017). Mothers may also experience stress if a child does not acquire adult roles, such as completing college, as this may violate normative expectations (Cepa & Furstenberg, 2021). Mothers are often emotionally invested in their adult children and report sadness, worry, or fear when adult children encounter challenges (Cichy et al., 2013). This may increase stress burden on mothers, manifesting in worse health outcomes.

Prior work establishes a connection between an adult child’s college completion and their aging mothers’ health. Drawing on data collected on older adults in Taiwan, Lee et al. (2017) found childrens’ education attainment shaped parents’ mental health trajectories such that adult childrens’ educational achievements reduced parents’ depressive symptoms. Yahirun et al. (2017) used data collected from older adults in Mexico to show childrens’ educational attainment was positively associated with parents’ physical functioning and overall longevity.

Several studies focus on the U.S., offering evidence for how childrens’ educational attainment shapes maternal health. Using the Within Family Differences Study focused on women ages 65–75 with at least two living children, Peng et al. (2019) examined how mothers’ mental health related to the proportion of children who completed college or more. A greater proportion of children who completed college reduced depressive symptoms and activity limitations, suggesting college completion benefitted aging mothers’ health (Peng et al., 2019). Yahirun et al. (2020) used Health and Retirement Study (HRS) data surveying adults 50 and older to examine the association between adult childrens’ education and parents’ mental health. They found childrens’ college completion reduced depressive symptoms among parents. Also using HRS data, Friedman and Mare (2014) found an adult child’s educational attainment reduced mortality risk for their parents, even when accounting for socioeconomic resources. To date, studies have not considered adult childrens’ college completion timing (on time, late, or never) on their mothers’ health, emphasizing the importance of timing of adult children’s events on mothers’ health.

### Adult Childrens’ College Completion Timing and Their Mothers’ Health

Adult childrens’ college completion timing may influence health of their aging mothers. For many young adults today, on time college completion falls around age 24 (Fomby & Bosick, 2013; Taniguchi, 2005). Prior work has not considered how timing may shape the association between adult childrens’ college completion and their aging mothers’ health, yet there is evidence to suggest timing may matter. Relative to completing college late or never, on time college completion suggests adult children may have earlier access to resources that accompany degree completion, including job stability, and higher income, among other benefits. In fact, Taniguchi (2005) found young adults who completed college at age 25 or later had significantly lower wage premiums relative to peers who completed college earlier. Another study using Italian university administrative and survey data from Alma Laurea Consortium found delayed college completion reduced employment probability and had a negative effect on monthly earnings (Aina & Casalone, 2020).

The benefits of on time college completion likely extend beyond adult children, shaping the health of aging mothers. Adult children who complete college on time may not only be less reliant on their mothers for support, but may also be able to provide their aging mothers with health resources (Oreopoulos & Petronijevic, 2013). On time college completion is considered a key marker of adulthood. When adult children complete college on time relative to late or never, their mothers may worry less about their adult children, thereby reducing stress and improving health (Cichy et al., 2013). Thus, we expect mothers of adult children who achieve a college degree on time will have less poor health relative to mothers of adult children who complete college late or who do not earn a college degree.

Since average college completion age falls around age 24, college completion at age 25 or later will be considered late college completion (Fomby & Bosick, 2013; Taniguchi, 2005). Although adult childrens’ late college completion may not have the same health benefits for aging mothers as on time college completion, it is likely that mothers with children who complete college late fare better than mothers with children who do not complete college. Relative to mothers who do not have adult children who complete college, mothers with adult children who finish college late may experience a reversal in flows of support such that mothers eventually provide less support to adult children, and adult children have more resources to offer their aging mother (Oreopoulos & Petronijevic, 2013). In addition, mothers may worry less about adult children even if they complete a degree later than expected (Cichy et al., 2013). Therefore, we predict mothers of adult children who complete college late will have better health than mothers of adult children who do not earn a college degree but will report poorer health than mothers of adult children who complete college on time.

### Variation by Maternal Educational Attainment

Prior work has called attention to divergent pathways into adulthood across socioeconomic backgrounds (Billari et al., 2019). With safety nets provided by parents, adult children of middle to upper socioeconomic backgrounds, operationalized through maternal educational attainment, often experience an elongated transition into adulthood, in which educational pursuits and exploration are encouraged (Cooper & Pugh, 2020). Conversely, adult children of lower socioeconomic background may experience “expedited adulthood,” in which adult children must establish independence and acquire roles symbolic of adulthood more quickly than their counterparts of a higher socioeconomic background (Cooper & Pugh, 2020).

Given socioeconomic variation in timing of entry into adult roles, mothers may have differing expectations about when adult children should complete college. To illustrate, Cepa and Furstenberg (2021) examined the importance and timing of adult milestones among Americans of lower versus higher socioeconomic background. Although they found consensus roles symbolic of adulthood, like education, they discovered individuals of a lower socioeconomic background expected adult roles to be achieved earlier than those of a higher socioeconomic background (Cepa & Furstenberg, 2021). Thus, mothers of a lower socioeconomic background may be more vulnerable to stress, and thus exhibit poorer health if their adult children do not complete college on time.

Prior research suggests mothers of a lower socioeconomic background whose adult children complete a college degree late or do not complete a college degree may face poorer health than mothers of a higher socioeconomic background whose adult children complete a college degree late or do not complete a college degree. Mothers of a lower socioeconomic background often experience “double jeopardy” in which they have fewer resources of their own but exert greater effort to aid struggling adult children, often offering time and other material resources when money is scarce (Fingerman et al., 2015). Yet, parents with less than a high school degree may experience the greatest health benefit from their adult child’s college completion (Yahirun et al., 2017). Based on this, we expect the association between adult childrens’ college completion timing and health to be stronger for mothers who have not completed a college degree.

### Additional Factors Related to Adult Childrens’ College Completion Timing and Their Mothers’ Health

In our study, we accounted for other factors linked to adult childrens’ college completion timing and their mothers’ health. We controlled for age of adult children and their mothers, as likelihood of college completion increases with age (NCES, 2022a), and age may influence mothers’ health evaluation (Jylhä, 2009). We also controlled for gender of the adult child, as previous research finds mothers report closer relationships to daughters over sons, suggesting daughters’ college completion timing may shape their mothers’ health more so than sons’ (Fingerman et al., 2020). We accounted for the adult child’s parenthood status, as mothers’ entrance into grandparenthood is linked to worse health (Wu et al., 2022). We included depressive symptoms of the adult children and their mothers, as previous research illustrates mental health is associated with physical health (Peleg & Nudelman, 2021). We also accounted for health of mothers from Wave I of AHPS, as earlier maternal health likely predicts future health. We also included health of adult children, given research supporting the intergenerational transmission of health (Duke et al., 2021). We accounted for adult childrens’ perceived closeness and communication frequency with their mother. Research suggests lower levels of mother–adult child closeness and communication are linked to worse physical health (Stepniak et al., 2020).

We controlled for whether the adult child ever married, divorced, or cohabited, since prior research has documented parents of adult children who never married or divorced had worse physical health relative to parents of adult children who were married (Zhang & Hammersmith, 2023). We account for adult childrens’ total siblings, as mothers’ number of children is linked to her health (Dior et al., 2013), with mothers who have two to four children reporting lowest mortality rates. We also included whether the child ever worked full time, as adult children who secure employment may provide greater financial resources to mothers (Gottlieb et al., 2014), potentially improving mothers’ health.

We included several characteristics of mothers, including race and ethnicity, as prior research documents greater emotional, instrumental, and financial support exchanges among Black, Latinx, and Asian families relative to white families (Zhang & Grant, 2023), but also barriers in completing college among Black and Latinx adults due to greater college debt (Houle et al., 2022). Black, Hispanic, and Asian mothers may also report poorer health due to experiences of economic, social, and institutional racism (Barr et al., 2018). We included mothers’ immigrant generation, as research suggests immigrant mothers may face barriers in health insurance access and report poorer health (Ro et al., 2022), but may also report greater family solidarity (Rumbaut & Komaie, 2010). Finally, we accounted for the mother’s full-time work, as this could be associated with the mothers’ available resources and her own health (Fingerman et al., 2015).

#### Hypotheses

Given the evidence provided above, we make three central predictions:

1. Mothers of adult children who complete college on time will have less poor health relative to mothers of adult children who complete a college degree late and mothers of adult children who do not earn a college degree.
2. Mothers of adult children who complete college late will have less poor health than mothers of adult children who do not earn a college degree and will have poorer health than mothers of adult children who complete college on time.
3. We expect the association between adult childrens’ college completion timing and health to be stronger among mothers without a college degree relative to mothers with a college degree.

## Research Design

**Fig. 1:**
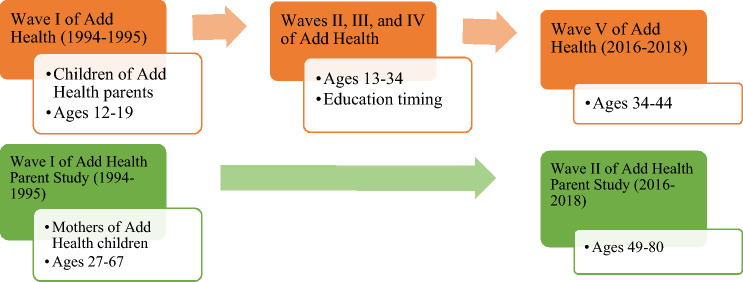
Data structure of add health and add health parent study for analytic sample

Data come from Waves I, III, IV, and V of Add Health and Wave I-II of AHPS (see Fig. 1). Add Health is a nationally representative sample of 20,745 adolescents in 7th–12th grades (12–21 years old) from 1994 to 1995 at Wave I (Harris et al., 2019). Respondents were followed up with Wave II in late adolescence from 1995–1996 (13–22 years old), Wave III in the transition to adulthood from 2001–2002 (18–28 years old), Wave IV in young adulthood from 2008–2009 (25–34 years old), and Wave V in adulthood from 2016–2018 (34–44 years old). In Add Health, Wave III corresponds to the transition to adulthood, Wave IV to young adulthood, and Wave V to adulthood (Harris et al., 2019). At Wave V, 12,300 respondents had completed the survey.

AHPS interviewed parents, predominantly mother Figs. (96%), of Add Health respondents from 1994–1995 at Wave I (AHPS Questionnaire) and from 2016–2018 at Wave II, with 2,244 parents completing the survey. The timing of Wave V of Add Health corresponds to Wave II of AHPS. We paired data for adult children who completed Waves I, III, IV, and V and had a mother who completed Wave II of AHPS, yielding a sample of 1,347 mothers after removing those with missing data on: Wave III (*n* = 357), Wave IV (*n* = 139), or Wave V (*n* = 332) completion and respondent’s gender (*n* = 1). We also excluded fathers (*n* = 47), as a majority of the sample (96%) were mother figures, and limited our sample to Black, Latinx, Asian, and white mothers due to small sample sizes for other groups (*n* = 21).

We used multiple imputation with chained equations in Stata 17.0 to infer missing data on independent variables (Royston & White, 2011). We imputed the independent and moderator variables: adult childrens’ college completion timing (*n* = 101) and mothers’ educational attainment at Wave I (*n* = 58). We also imputed control variables of adult children including whether they ever cohabited by Wave IV (*n* = 4), their health at Wave IV (*n* = 1), gender at Wave I (*n* = 1), and frequency of communication with their mother (*n* = 203). For mothers, we imputed depressive symptoms at Wave II (*n* = 6) and health at Wave I (*n* = 18).

### Independent Variables

*College Completion Timing*. Adult childrens’ college completion timing included the categories: 1) never completed; 2) on time completion; and 3) late completion. Using work by Fomby and Bosick (2013) and Taniguchi (2005), adult children who completed college before age 25 were considered on time completers, and those who completed college at age 25 or later were considered late completers. Those who had not completed college were in the never completed category. These measures were drawn from questions regarding college completion timing from Waves III, IV, and V. At Wave III, adult children were asked: “In what month and year did you receive your bachelor’s degree?” The adult child’s age that they completed college was created by subtracting their birth year from the year they received a bachelor’s degree. At Wave IV, adult children were asked “What is the highest level of education that you have achieved to date” and “In what year did you receive your most recent degree?” If the adult child completed college at Wave IV, completion age was created by subtracting their birth year from the year they received a bachelor’s degree. At Wave V, college completion timing was not asked. Instead, we used the question: “What is the highest level of education that you have achieved to date?” Adult children who never completed college by Wave V were never-completers; adult children who completed college before age 25 were on time completers, and those who completed college after age 25 or after Wave IV were late completers.

### Dependent Variable

*Mothers’ Self-Rated Health.* We included a dichotomous measure of mothers’ self-rated health at Wave II of AHPS from the question: “Would you say your health is excellent, very good, good, fair, or poor?” Responses ranged from excellent (1), very good (2), good (3), fair (4), and poor (5). Given the distribution of responses was not unimodal, we collapsed responses into two categories: good self-rated health (good, very good, and excellent health, coded 0) and poor self-rated health (fair and poor health, coded 1); (Manor et al., 2000).

### Moderator

*Mothers’ Educational Attainment.* Socioeconomic background was operationalized using mothers’ educational attainment, specifically whether they completed at least a college degree (1) or not (0) at Wave I of the AHPS, ensuring mothers completed college prior to adult children.

### Controls

Child’s age was determined from their age at Add Health Wave IV. Gender measured whether the adult child identified as man (1) or woman (2) at Wave I. Add Health did not include a non-binary gender category at Wave I. Depressive symptoms at Wave IV of Add Health and Wave II of AHPS (α = 0.78) were based on the CES-D scale on whether the adult child and mother felt bothered, could not shake off the blues, felt just as good as others (reverse-coded), had trouble keeping mind, felt depressed, felt too tired to do things, and felt sad in the past 7 days. Responses ranged from 0 (not at all) to 3 (most of the time or all the time). Communication frequency with the adult child’s mother came from the Wave IV question: “How often do you and your [mother figure] talk on the telephone, exchange letters, or exchange email?” Responses ranged from never (0), once a year or less, (1), a few times a year (2), once or twice a month (3), once or twice a week (4), to almost every day (5). Adult child’s perceived closeness to their mother came from the Wave IV question, “how close do you feel to your mother figure?” Responses ranged from not at all close (1), not very close (2), somewhat close (3), quite close (4), to very close (5).

Whether the adult child ever married by Wave IV came from the question: “How many persons have you ever married?” Responses were dichotomized to 0 (never married) and 1 (ever married). Whether the adult child ever divorced by Wave IV came from the question: “How did your marriage end?” If the adult child responded with “divorce,” the response was coded as 1 and 0 otherwise. Whether the adult child ever cohabited came from a Wave IV question: “How many romantic or sexual partners have you ever lived with for one month or more?” Responses greater than 0 were coded 1 and 0 otherwise. Whether the adult child was a parent by Wave IV counted whether they had at least one child at the time of interview. Adult child’s number of siblings came from a Wave IV question: “How many brothers and sisters do you have, both living and deceased? Include biologically related, adoptive, and stepbrothers or sisters.” Whether the adult child ever worked full time at Wave IV came from the question, “Have you ever worked full time at least 35 h a week at a paying job while you were not primarily a student? Do not include summer work.” Responses included no (0) and yes (1). The adult child’s self-rated health was derived from the Wave IV question: “In general, how is your health?” Responses included 1 (excellent), 2 (very good), 3 (good), 4 (fair), and 5 (poor), which were dichotomized into good (excellent, very good, and good, coded 0) and poor self-rated health (fair and poor, coded 1).

Mothers’ race and ethnicity came from the questions asked at Wave II of AHPS: “What is your race?” and “Are you of Hispanic or Latino origin?” From those two questions, responses included non-Latinx Black, Latinx, non-Latinx Asian, and non-Latinx white. Mothers’ immigrant generation came from the question: “Were you born a U.S. citizen?” Responses included no (0) and yes (1). Mothers’ employment came from the question: “Please select all that apply to your current employment situation: working for pay.” Responses included no (0) and yes (1). Mothers’ age was from AHPS Wave II. We included self-rated health of the adult child’s mother at Wave I of AHPS, using the question: “How is your general self-rated health?” Responses ranged from (1) excellent, (2) very good, (3) good, (4) fair, and (5) poor, which were dichotomized into good (excellent, very good, and good, coded 0) and poor self-rated health (fair and poor, coded 1).

### Analytic Strategy

First, we generated summary statistics of variables for the adult child and their mother for the overall sample, and by mothers’ educational attainment (less than college degree relative to college degree or more). To answer our first research question, on whether adult childrens’ college completion timing was associated with their mothers’ health, we ran logistic regressions to examine associations between college completion timing by adult children and their mothers’ health. To answer our second research question, on whether the association between adult childrens’ college completion timing and their mothers’ self-rated health varied across maternal educational attainment, we ran logistic regression models including interactions of mothers’ educational attainment and adult childrens’ college completion timing.

We also ran supplemental analyses restricting the sample to Add Health mothers who had multiple children who completed the Add Health survey, to assess whether these associations persisted across various siblings’ college completion timing (*n* = 322). All analyses were weighted using AHPS survey weights in Stata 17.0 to ensure results were nationally representative (Chen & Harris, 2020).

## Results

Summary statistics for adult children are displayed in Table 1, for the full sample, and by mothers’ educational attainment. In the full sample, over half (55%) were women. On average, respondents were 29 years old at Wave IV and 38 years old at Wave V. Nearly 80% of adult children were parents by Wave IV. At Wave IV, on average, adult children experienced fewer than one depressive symptom per week (0.52 symptoms). Adult childrens’ frequency of communication and closeness with mothers were high. For college completion timing, most adult children had never completed college (62%), followed by 25% who graduated college on time, and 13% who graduated college late.

**Table 1:** Summary statistics for adult children by their mothers’ educational attainment

Variable	Full sample		Maternal Ed: < College		Maternal Ed: College +		Range	Alpha
	Mean	SE	Mean	SE	Mean	SE		
Age of adult child at Wave IV	28.64	0.05	28.68	0.07	28.53	0.10	25 to 34	
Had child by young adulthood	0.80	–	0.91	0.04	0.50*	–	0 to 1	
Ever divorced by Wave IV	0.02	–	0.03	0.01	0.01	–	0 to 1	
Ever cohabit by Wave IV	0.49	–	0.51	0.02	0.43*	–	0 to 1	
Ever married by Wave IV	0.51	–	0.52	0.02	0.46	–		
Adult child’s number of siblings at Wave IV	2.46	0.06	2.60	0.07	2.09*	0.10	0 to 20	
Ever worked at Wave IV	0.96	–	0.96	0.01	0.96	–	0 to 1	
Self-rated health of adult child at Wave IV	0.07	0.01	0.04	0.01	0.08*	0.01	0 to 1	
CES-D scale at Wave IV	0.52	0.01	0.55	0.01	0.46*	0.02	0 to 3	0.78
Frequency of communication with mother at Wave IV	4.33	–	4.34	0.04	4.30	–	0 to 5	
Maternal closeness of adult child at Wave IV	4.54	–	4.53	0.03	4.56	–	1 to 5	
College completion timing							0 to 2	
Never	0.62	–	0.69	0.02	0.41*	–	1 to 3	
On time (before age 25)	0.25	–	0.19	0.01	0.42*	–		
Late (ages 25 +)	0.13	–	0.12	0.01	0.16	–		
*n*	1347		938		409			

Young adulthood corresponds to Wave IV of Add Health, and adulthood corresponds to Wave V of Add Health

*Significant difference from mothers with less than college degree at p < 0.05-level

Significantly more mothers who had less than a college degree had children who never completed college relative to mothers with a college degree (69% v. 41%). Mothers with a college degree were more likely to have children complete college on time relative to mothers without a college degree (42% v. 19%).

Table 2 shows summary statistics of mothers from AHPS, for the full sample and by mothers’ educational attainment. All mothers identified as women (100%). Mothers identified as non-Latinx white (78%), non-Latinx Black (11%), Latinx (9%), and non-Latinx Asian (2%). Mothers were in midlife through older adulthood and were approximately 63 years old, with ages ranging from 49 (midlife) to 80 (old age). About 94% were born in the U.S and most reported less than a college degree (73%). Mothers reported 1.42 depressive symptoms on the CES-D scale, or a little over one symptom of depression per week. About 11% of mothers reported poor health in Wave I of AHPS. By Wave II, this had more than doubled with 28% of mothers reporting poor health.

**Table 2:** Summary statistics for add health mothers by their mothers’ educational attainment

Variable	Full sample		Maternal ed: < College		Maternal ed: College +		Range	Alpha
	Mean	SE	Mean	SE	Mean	SE		
Age of respondent	62.61	0.16	61.97	0.20	64.27*	0.28	49 to 80	
Immigrant generation status							0 to 1	
1st generation	0.06	–	0.07	0.01	0.95	–		
2nd + generation	0.94	–	0.93	0.01	0.05	–		
Employment status							0 to 1	
Not employed	0.60	–	0.59	0.02	0.64	–		
Employed	0.40	–	0.41	0.02	0.36	–		
Self-rated health of mother at Wave I	0.11	0.01	0.13	0.01	0.05*	0.01	0 to 1	
CES-D Scale at Wave II	1.42	0.01	1.46	0.02	1.30*	0.02	1 to 4	0.79
Self-rated health of mother at Wave II	0.28	0.01	0.33	0.02	0.16*	0.02	0 to 1	
College completion at Wave I							0 to 1	
Less than a college degree	0.72	–	1.00	0.00	0.00*	–		
College degree and more	0.28	–	0.00	0.00	1.00*	–		
*n*	1347		938		409			

*Significant difference from mothers with less than college degree at p < 0.05-level

By mothers’ educational attainment, mothers who completed college were slightly older, had fewer depressive symptoms relative to mothers who completed less than a college degree, and reported better health.

Table 3 shows coefficients and odds ratios from the logistic regression of the association of adult childrens’ college completion timing on their mothers’ health, with the reference category of adult children who completed college on time (results displaying controls available upon request). We conducted stepwise regression models, first without controls (Model 1), then including mothers’ and adult childrens’ controls (Model 2) and adding interactions of childrens’ college completion timing and mothers’ educational attainment (Model 3).

**Table 3:** Logistic regression of adult childrens’ college completion timing predicting maternal self-rated health including moderation by mothers' education (reference category: on time college completion)

Adult childrens’ college completion timing	Basic (1)				Controls (2)				Interactions (3)			
	Coeff	SE		OR	Coeff	SE		OR	Coeff	SE		OR
Never	1.09	0.19	***	2.97	0.77	0.24	**	2.16	0.70	0.25	**	2.01
Late	0.38	0.26		1.46	0.14	0.30		1.15	0.17	0.33		1.19
Mother completed college at Wave I					−0.52	0.21	*	0.59	−0.65	0.37		0.52
Age of mother at Wave II					−0.03	0.02		0.97	−0.03	0.02		0.97
College completion timing and interactions with maternal education												
Never completed college $\times$ Mother completed college									0.25	0.46		1.28
Completed college late $\times$ Mother completed college									−0.17	0.63		0.84
Constant	−1.70	0.17	***	0.18	−3.16	1.79		0.04	−3.13	1.79		0.04
F-statistic	19.23				7.92				7.29			
N	1347				1329				1329			

Coeff. Stands for coefficient. OR stands for odds ratio. SE stands for standard error. The reference category for timing of educational transitions is completed college on time. We controlled for the following variables: race and ethnicity of the mother, immigrant generation status of the mother, employment status of the mother, CES-D scale of mother at Wave II, self-rated health of the mother at Wave I, gender of the adult child, age of the adult child, whether the child was a parent by Wave IV, whether the adult child had ever divorced, ever married, and ever cohabited by Wave IV, the adult child's number of siblings, the adult child's self-rated health and CES-D scale, frequency of communication with their mother, and maternal closeness of adult child. The reference category for the race and ethnicity of the parent is Non-Latinx white. The reference category for immigrant generation status is born in the United States. The reference category for maternal education is did not complete college. The reference category for the gender of the respondent is male. The reference category for being a parent by Wave IV was not a parent by Wave IV. The reference category for ever divorced by Wave IV was never divorced by Wave IV. The reference category for ever cohabited by Wave IV was never cohabited by Wave IV

*p < 0.05. ** p < 0.01. ***p < 0.001

In the basic model (Model 1), mothers of adult children who never completed college had significantly greater odds of reporting poorer health (2.97 times) relative to mothers of adult children who completed college on time. Though mothers of adult children who completed college late had greater odds of reporting poorer health relative to mothers of adult children who completed college on time, this association was not statistically significant.

When including controls (Model 2), associations remained similar. Mothers of adult children who never completed college had significantly greater odds of reporting poor health (2.16 times) relative to mothers of adult children who completed college on time. Mothers of adult children who completed college late had a greater likelihood of reporting poor health, though this association was not significant. Mothers who completed college at Wave I of the AHPS, when their children were adolescents from 1994–1995, had significantly lower odds of reporting poor health relative to mothers who did not complete college at Wave I of the AHPS.

In Model 3, we included interactions of mothers’ educational attainment and adult childrens’ college completion timing. Mothers of adult children who had never completed college had significantly greater odds of reporting poorer health (2.01 times) relative to mothers of adult children who completed college on time. Thus, we only found support for Hypothesis 1, that mothers of adult children who completed college on time would have better health than mothers of adult children who never completed college. However, we did not find evidence to support Hypothesis 2, that mothers of adult children who completed college on time would have better health than mothers of adult children who completed college late. Upon including interactions of mothers’ educational attainment and of adult childrens' college completion timing, no significant findings emerged, lending no support for Hypothesis 3, or the association between college completion timing and health was not stronger among mothers without a college degree relative to mothers who completed college.

In supplemental analyses, we restricted the sample to mothers (*n* = 322) of adult children who had at least one other sibling who also completed Add Health Wave V (results available upon request), to examine whether varying siblings’ education timing was associated with maternal health. Our results were consistent with findings from the full sample in Table 3. Supporting Hypothesis 1, in the model with all controls, mothers of children who never completed college had significantly greater odds (1.49 times) of reporting poor health relative to mothers of adult children who completed college on time. Mothers of children who completed college late had greater odds of reporting poor health (0.84 times), though this was not significant. We did not find mothers’ educational attainment moderated the association between adult childrens’ college completion timing and maternal health.

Table 4 shows coefficients and odds ratios from the logistic regression of adult childrens’ college completion timing on their mothers’ health, with adult children who completed college late as the reference category (results with controls available upon request).

**Table 4:** Logistic regression of adult childrens’ college completion timing predicting maternal self-rated health including moderation by mothers' education (reference category: late college completion)

Adult childrens’ college completion timing	Basic Model (1)				Controls (2)				Interactions (3)		
	Coeff	SE		OR	Coeff	SE		OR	Coeff	SE	OR
Never	0.71	0.22	**	2.03	0.63	0.25	*	1.88	0.53	0.28	1.70
On time	−0.38	0.26		0.68	−0.14	0.30		0.87	−0.17	0.33	0.84
Mother completed college at Wave I					−0.52	0.21	*	0.59	−0.82	0.50	0.44
Age of mother at Wave II					−0.03	0.02		0.97	−0.03	0.02	0.97
College completion timing and interactions with maternal SES											
Never completed college $\times$ Mother completed college									0.42	0.57	1.52
On time college completion $\times$ Mother completed college									0.17	0.63	1.19
Constant	−1.32	0.20	***	0.27	−3.03	1.77		0.05	−2.96	1.77	0.05
F-statistic	19.23				7.92				7.29		
N	1347				1329				1329		

Coeff. Stands for coefficient. OR stands for odds ratio. SE stands for standard error. The reference category for timing of educational transitions is completed college late. We controlled for the following variables: race and ethnicity of the mother, immigrant generation status of the mother, employment status of the mother, CES-D scale of mother at Wave II, self-rated health of the mother at Wave I, gender of the adult child, age of the adult child, whether the child was a parent by Wave IV, whether the adult child had ever divorced, ever married, and ever cohabited by Wave IV, the adult child's number of siblings, the adult child's self-rated health and CES-D scale, frequency of communication with their mother, and maternal closeness of adult child. The reference category for the race and ethnicity of the parent is Non-Latinx white. The reference category for immigrant generation status is born in the United States. The reference category for maternal education is did not complete college. The reference category for the gender of the respondent is male. The reference category for being a parent by Wave IV is was not a parent by Wave IV. The reference category for ever divorced by Wave IV was never divorced by Wave IV. The reference category for ever cohabited by Wave IV was never cohabited by Wave IV

*p < 0.05. ** p < 0.01. ***p < 0.001

In Model 1, mothers whose adult children never completed college had significantly greater odds of reporting poor health (2.03 times) relative to mothers with adult children who completed college late. On the other hand, mothers whose adult children completed college on time had lower odds of reporting poor health (0.32 times), relative to mothers with adult children who completed college late. However, this was not significant.

Including mothers’ and adult childrens’ controls in Model 2, mothers whose adult children never completed college had significantly greater odds of reporting poor health (1.88 times) relative to mothers of adult children who completed college late. Mothers whose adult children completed college on time had lower odds of reporting poor health (0.13 times), relative to mothers of adult children who completed college late. Mothers’ age was significantly associated with their health.

In Model 3, we included interactions between mothers’ educational attainment and adult childrens’ college completion timing. Though none reached statistical significance, like Models 1 and 2, mothers of adult children who never graduated college had greater odds of reporting poor health (1.70 times), relative to mothers of adult children who completed college late. Mothers of adult children who completed college on time had lower odds of reporting poor health relative to mothers of adult children who completed college late (0.16 lower). However, we did not find mothers of adult children who completed college late had significantly poorer health relative to mothers of adult children who completed college on time.

Our analyses revealed partial support for Hypothesis 2. Having an adult child who completed college late relative to an adult child who never finished college was associated with less poor health for mothers. Yet, we found no difference between mothers of adult children who completed college on time relative to mothers of adult children who completed college late. Adding interactions between mothers’ educational attainment and adult childrens’ college completion timing did not yield significant findings, lending no support for Hypothesis 3, or the association between adult childrens’ college completion timing and health would be stronger among mothers without a college degree relative to mothers with a college degree.

In supplemental analyses, we restricted the sample to mothers (*n* = 322) of adult children who had at least one other sibling who also completed Add Health Wave V survey (results available upon request), to examine whether varying siblings’ education timing was associated with maternal health. Our results were consistent with findings from the full sample (Table 4). In the model with all controls, mothers of adult children who never completed college had significantly greater odds (0.65 times) of reporting poor health relative to mothers of adult children who completed college late. Mothers of adult children who completed college on time had lower odds of reporting poor health (0.84 times) relative to mothers of adult children who completed college late, though this was not statistically significant. As in Table 3, we did not find mothers’ educational attainment moderated the association between adult childrens’ college completion timing and maternal health.

## Discussion

As the lifespan of mothers has increased, mothers and adult children depend more on each other for instrumental, emotional, and financial support (Barr et al., 2018). In this study, we examined whether adult childrens’ college completion timing was associated with their mothers’ health using nationally representative data from Add Health and AHPS. Our results showed mothers whose adult children completed college on time or late had less poor health relative to mothers whose adult children never completed college by adulthood. These findings support life course perspective (Elder, 1998)—mothers’ health was associated with adult childrens’ college completion timing, reinforcing the concept of *linked lives*. Nonetheless, we did not find the association between a child’s college completion and mothers’ health was conditioned on maternal education. The results can be categorized into two key themes: adult childrens’ college completion, regardless of timing, did indeed shape their mothers’ health, and the returns of adult childrens’ college completion on mothers’ health did not vary by mothers’ educational attainment.

### Adult Childrens’ College Completion Timing and Their Mothers’ Health

The first theme was that adult childrens’ college completion, if completed on time or late, was associated with less poor health for mothers. Today, young adults spend more years pursuing higher education (2022b; NCES, 2022a). In accordance with the life course principle, *linked lives* (Elder, 1998)*,* college completion delays will not only shape the life of the adult child, but also health and well-being of their mother by potentially extending the time mothers provide financial, instrumental, and emotional support to adult children. In turn, this may heighten mothers’ stress if their child does not complete college, in that it may increase the mother’s support burden (Barr et al., 2018; Yahirun et al., 2020). College completed on time or late compared to never may lead to greater financial returns not only for adult children, but their mothers, from increased health-promoting information and exchanges of resources (Friedman & Mare, 2014).

Though we expected mothers of adult children who completed college late would report poorer health than mothers of adult children who completed college on time, we found mothers of adult children who completed college on time and late had less poor health than mothers of adult children who had never completed college by adulthood, even when restricting the sample to mothers with multiple adult children represented in Add Health. Nonetheless, this finding still supports *linked lives*, or circumstances in the adult child’s life (e.g., college completion) can shape the health of their mother (Elder, 1998). Moreover, this finding aligns with the previous research showing parents’ well-being is dependent on adult childrens’ college completion (Lee et al., 2017; Peng et al., 2019; Yahirun et al., 2020). These results show given delays in the transition to adulthood, especially with regard to college completion, mothers’ health may not be at risk if their adult childrens’ degree is completed on time or late. Although we did not find a difference in health for mothers by their adult child’s college completion timing, it is possible an adult child’s delayed college completion could result from conflicting demands, such as offering financial or time support to an aging mother. Caring for an aging mother could postpone one’s college completion, while also benefiting health of the mother, possibly washing out an effect of late college completion.

### Variation by Maternal Educational Attainment

The second theme was that health returns for adult childrens’ college completion did not vary by their mothers’ educational attainment. Based on prior work, we expected to find mothers’ educational attainment would shape adult childrens’ completion of college. Our results did show adult children of mothers who completed college were more likely to be college graduates while adult children with mothers who did not complete college were less likely to earn a college degree. These descriptive findings provide some evidence for “expedited adulthood” among individuals of a lower socioeconomic background—adult children of a lower socioeconomic background may forgo college altogether to enter the workforce relative to peers who experience an “elongated” pathway into adulthood (Cooper & Pugh, 2020).

Nonetheless, we did not find differences in mothers’ health by educational attainment for the association between adult childrens’ college attainment timing and mothers’ health. This conflicts with the findings from prior research. Fingerman and colleagues (2015) found a “double jeopardy” effect, or mothers with fewer socioeconomic resources go above and beyond to assist adult children, even if their own financial resources are scarce. This means, mothers of a lower socioeconomic background may suffer greater health consequences when trying to help adult children succeed. Yet, Yahirun et al. (2017) found parents with less than a high school degree often experience the greatest health benefit when adult children achieve educational success. Our null findings might be attributed to mothers’ expectations of adult children. In a 2019 study, Billari and colleagues found support for “stratified socialization,” or mothers of a lower socioeconomic background may support childrens’ early adoption of adult roles (e.g., forgoing college), as a way to establish independence earlier (Billari et al., 2019). Given this “expedited” pathway into adulthood for children, mothers of a lower socioeconomic background may have adult children who have achieved independence earlier in their lives, therefore no longer needing to offer continued support to adult children, and thus, face fewer corresponding health repercussions, compared to mothers of adult children of a higher socioeconomic background, who may need to offer continued support to adult children to complete adult milestones.

### Limitations and Future Directions

Although these findings inform how adult childrens’ college completion timing relates to health for their mothers, there were several limitations to this study. First, we could not make causal inferences from our analyses, so our results may be affected by omitted variable bias, in that mothers’ characteristics may predict adult childrens’ ability to complete their degree, which may in turn shape mothers’ health.

Second, there were also no direct measures of worries about adult children in AHPS, making it difficult to assess how mothers feel about the child and their transition into adulthood. Future work should consider how mechanisms, like those related to parent–child relationship quality, may shape the relationship between adult childrens’ college timing and their mothers’ health. We could not include measures of caregiving, and thus could not assess the role of care provided to aging mothers in the relationship between adult childrens’ degree completion timing and their mothers’ health. Mothers’ college attainment was a proxy for socioeconomic background, as over 40% of responses on income within the analytic sample of AHPS were missing. If possible, studies should use parental income or wealth as an indicator of socioeconomic background when considering the relationship between an adult childrens’ degree completion timing and their mothers’ health.

Due to the small sample size and sampling strategy of AHPS, we omitted fathers. Mothers often share closer relationships with children as they age (Fingerman et al., 2020). Thus, we expect differences in how adult childrens’ college completion timing shapes health of aging fathers relative to mothers. Future research should examine how adult childrens’ college timing differentially relates to mothers’ and fathers’ health. Last, due to a small sample of Black, Hispanic, and Asian parents in AHPS, we were unable to disaggregate analyses by race and ethnicity. Given that racial disparities in the completion of a college degree persist in the United States (2022b; NCES, 2022a), future research should examine the relationship between adult childrens’ college completion timing and parents’ mental and physical health, examining variation across racial and ethnic background. Through this, future work may better assess how and whether adult childrens’ college timing shapes health differentially across racial and ethnic groups, and may be a greater protective factor for some groups rather than others.

## Conclusion

Adult childrens’ college completion, regardless of timing, was associated with their mothers’ improved health. Although differences in mothers’ health by adult child’s college completion timing did not emerge, future work should examine whether degree attainment timing matters in shaping the child’s trajectory into adulthood. As college completion is often a precursor to other adult roles (Cepa & Furstenberg, 2021), researchers, policymakers, and practitioners should evaluate how college completion may shape timing of other markers of adulthood such as employment, marriage, and childbearing. More importantly, it is worth considering how co-occurring or subsequent delays in role attainment may shape aging mothers’ health. Therefore, programs and policies facilitating adults’ college completion may have positive impacts on mothers’ health.
